# Lifetime affective problems and later-life cognitive state: Over 50 years of follow-up in a British birth cohort study

**DOI:** 10.1016/j.jad.2018.07.078

**Published:** 2018-12-01

**Authors:** Sarah-Naomi James, Daniel Davis, Celia O'Hare, Nikhil Sharma, Amber John, Darya Gaysina, Rebecca Hardy, Diana Kuh, Marcus Richards

**Affiliations:** a MRC Unit for Lifelong Health and Aging at UCL, 33 Bedford Place, WC1B 5JU, London, United Kingdom; b EDGE Lab, School of Psychology, University of Sussex, BN1 9RH, Brighton, United Kingdom

**Keywords:** Depression, Anxiety, Affective, Cognition, Cognitive state, Life course, ACE-III, Addenbrooke's Cognitive Examination third edition, CI, confidence interval, GHQ-28, 28-item General Health Questionnaire, MRC, Medical Research Council, NSHD, National Survey of Health and Development

## Abstract

**Background:**

Affective problems increase the risk of dementia and cognitive impairment, yet the life course dimension of this association is not clearly understood. We aimed to investigate how affective problems across the life course relate to later-life cognitive state.

**Methods:**

Data from 1269 participants from the Medical Research Council National Survey of Health and Development (NSHD, the British 1946 birth cohort) were used. Prospectively-assessed measures of affective symptoms spanning ages 13–69 and categorised into case-level thresholds. Outcomes consisted of a comprehensive measure of cognitive state (Addenbrooke's Cognitive Examination (ACE-III)), verbal memory, and letter search speed and accuracy at age 69.

**Results:**

Complementary life course models demonstrated that having 2 or more case-level problems across the life course was most strongly associated with poorer cognitive outcomes, before and after adjusting for sex, childhood cognition, childhood and midlife occupational position and education.

**Limitations:**

A disproportionate loss to follow-up of those who had lower childhood cognitive scores may have led to underestimation of the strength of associations.

**Discussion:**

Using a population-based prospective study we provide evidence that recurrent lifetime affective problems predicts poorer later-life cognitive state, and this risk can be already manifest in early old age (age 69). Our findings raise the possibility that effective management to minimise affective problems reoccurring across the life course may reduce the associated risk of cognitive impairment and decline.

## Highlights

• Recurrent lifetime affective problems predict diminished late-life cognitive state.
• Those with affective problems only once do not show risk of lower cognitive state.
• Recurrence, rather than timing, of problems is more predictive.
• These associations remain even when controlling for prior childhood cognition.
• The risk of lower cognitive state is already manifest in early old age (age 69).

## 1 Introduction

Many studies have demonstrated an association between depression and anxiety – affective symptoms – and subsequent cognitive impairment and dementia (Cherbuin et al., 2015, da Silva et al., 2013, Gulpers et al., 2016, John et al., 2018, Jorm, 2001, Ownby et al., 2006, Stella et al., 2014). The severity, frequency and onset of symptoms are thought to be important features in establishing the nature of these associations (Bennett and Thomas, 2014, Byers and Yaffe, 2011, da Silva et al., 2013, Kaup et al., 2016, Köhler et al., 2010, Richards et al., 2014, Singh-Manoux et al., 2017), and feasibly affect cognitive function before dementia onset (Brailean et al., 2008, Butters et al., 2008, Luppa et al., 2013), perhaps through hippocampal atrophy (MacQueen and Frodl, 2011, McKinnon et al., 2009); although findings for cognitive decline are more inconsistent (Brailean et al., 2017). However, few studies have had long-term follow-up of affective symptoms and thus little is known about the life course accumulation of affective symptoms, and the relevance of symptom timing, in relation to later-life cognitive state (Richards et al., 2014, Riddle et al., 2017).

Using the Medical Research Council (MRC) National Survey of Health and Development (NSHD) – the British 1946 birth cohort – no clear pattern of association between longitudinal profiles of affective symptom trajectories derived from latent class analysis (aged 13–53 years) and level and change in cognitive test scores at ages 53 and 60–64 was found, even after adjusting for childhood cognitive ability, education and midlife socioeconomic position (Richards et al., 2014). However, participants may have still been relatively young; the symptom profiles did not allow the investigation of timing effects; and the cognitive outcomes may not have sufficiently captured aspects of function relevant to dementia risk (Richards et al., 2014). A new wave of cognitive data has now been collected on participants at age 69, repeating the cognitive function tests used previously, and adding one of the most detailed measure of cognitive state, the Addenbrooke's Cognitive Examination third edition (ACE-III) (Hsieh et al., 2013). Using complementary life course models can help to test the cumulative and temporal effects of affective symptoms on later-life cognitive state.

The aim of the present study was to investigate the cumulative association between case-level affective problems measured from adolescence to later-life and later life cognitive function at age 69, after accounting for sex, childhood cognition, educational attainment and lifetime socioeconomic position. We further aimed to investigate temporal effects of symptom occurrence by examining the incidence of affective problems in later life compared with earlier life, testing whether cumulative or time period life course models best described the data. We hypothesised that having more case-level affective problems across the life course would be associated with lower cognitive function in older age, independently of these potential confounders.

## 2 Methods

### 2.1 Participants

The NSHD is a representative sample of 5362 males and females who were born in England, Scotland and Wales in one week in March 1946 (Wadsworth et al., 2006). The 24th data collection was conducted between 2014 and 2015 when participants were aged 68–69 years (Kuh et al., 2016). At age 69, following a postal questionnaire at age 68, participants still alive and with a known current address in mainland Britain (*n* = 2698) were invited to have a home visit; 2149 (79%) completed a visit (see Supplementary Fig. I for a flow diagram of the sample). For this data collection, we obtained ethical approval from the NRES Queen Square REC (14/LO/1073) and Scotland A REC (14/SS/1009). All participants gave written informed consent to collect these data. Research was conducted in accordance with the Helsinki Declaration.

### 2.2 Cognitive outcomes

The ACE-III, a test of cognitive state (Hsieh et al., 2013), was used as the primary outcome measure. The ACE-III is divided into five domains: attention and orientation (scored 0–18); verbal fluency (0–14); memory (0–26); language (0–26); and visuospatial function (0–16). Thus the maximum total score is 100. A customised version of the ACE-III was administered by iPad using ACEMobile (http://www.acemobile.org); where this was not possible, a paper version was used. All offline scoring was undertaken by trained personnel. Of the 2149 participants with a home visit at age 69, 32 refused or were unable to undertake the ACE-III at all. Of the remaining 2117, 35 attempted but did not fully complete due to equipment error and inability to complete all sections and data from 353 participants were corrupt through equipment failure such as exporting data and the fieldwork agency being unable to retrieve the data from the iPad. Thus complete ACE-III data were available for 1729 participants, 81% of those who received home visit.

Of the 2149 participants with a home visit, 2102 (98%) completed a short-term verbal memory test and a processing speed test previously given at ages 43, 53 (Richards et al., 2004) and 60–64 years (Richards et al., 2014). The verbal memory test consisted of a 15-item word learning task devised by the NSHD. Similar to previous analyses (Richards et al., 2014), the total number of words correctly recalled over three identical trials was summed to provide an overall score for short-term verbal memory (maximum 45). Processing speed was assessed by a visual search task, where participants were required to cross out the letters P and W, randomly embedded within a page of other letters, as quickly and accurately as possible within 1 min. Letter search speed was represented by the position reached at the end of this interval (maximum 600) and letter search accuracy was represented by the number of target letters correctly crossed out within this interval (maximum 84). The degree of cognitive decline in the cohort using these measures has previously been described (Davis et al., 2017).

### 2.3 Lifetime affective symptoms measures

Due to the nature of data collection across the entire lifespan, different assessments of affective symptoms were necessary at specific ages. In order to use the most clinically meaningful metric at each age we identified those with a level of symptom severity consistent with a possible clinical diagnosis of affective disorder, referenced as those with “case-level symptoms”. More detailed information about the measures and validation of cut-off thresholds to indicate case-level symptoms can be found in Supplementary Table I. Briefly, at ages 13 and 15 years teacher ratings of behaviour and temperament were obtained using a forerunner of the Rutter A scale (Rutter, 1967). Factor scores at ages 13 and 15 years were summed to create scales representing a dimension of emotional problems, and were standardised to a mean of 0 and SD of 1. Frequency and severity of common symptoms of depression and anxiety were also assessed in adulthood, with the short community version of the Present State Examination at 36 years (Wing et al., 1974), the Psychiatric Symptom Frequency scale at 43 years (Lindelow et al., 1997), and the 28-item General Health Questionnaire (GHQ-28) (Goldberg and Hillier, 1979) at ages 53, 60–64, and 69 years. In line with previous studies (Hatch et al., 2009), for each total score thresholds for case-level symptoms were imposed, representing potentially diagnosable common mental disorder. For the adolescent teacher ratings this was the 91st to 100th percentile (Colman et al., 2007). For the Present State Examination this was the standard Index of Definition ≥ 5. For the Psychiatric Symptom Frequency scale this was greater than 22 (Lindelow et al., 1997) which has been shown to capture service contact for common mental disorders, relevant medication prescription and suicidal ideation. The threshold for the GHQ-28 was the recommended 4/5 cut for summed scores each recoded from the Likert scale to the binary scale (Goldberg and Hillier, 1979). The number of times participants met case threshold across testing waves were summed and recoded as follows: (a) never case-level (*n* = 764); (b) once only (*n* = 464); (c) twice or more (*n* = 313). Case level frequencies are shown in Supplementary Table II.

**Fig. 1:**
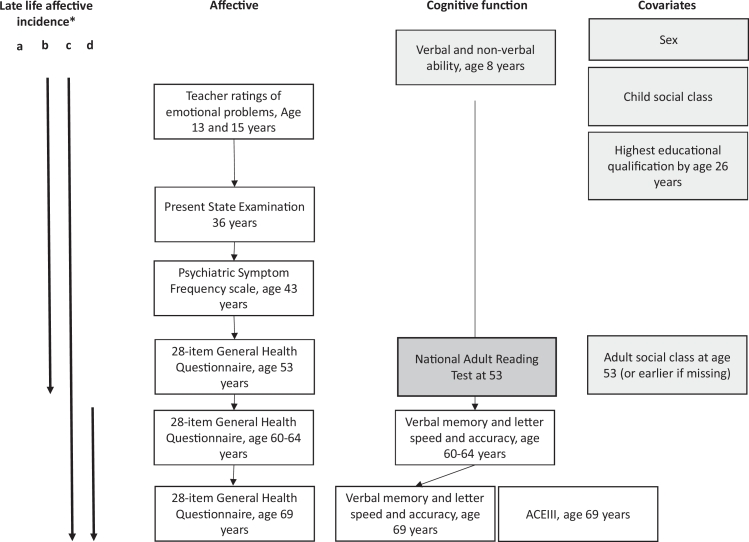
Flow diagram for MRC National Survey of Health and Development data used in this study*. Grey boxes = additional adjustments in model 2, Dark grey = additional adjustments in model 3. *Late-life incidence categories (a) Never case-level (b) Case-level in earlier-life only (age ≤ 53) (c) Case-level incidence in later-life (age ≥ 60) and in earlier-life (age ≤ 53) (d) first incidence of case-level in late-life (age ≥ 60).

To investigate effects of later incidence (age 60+) of case-level affective problems compared to earlier incidence (<age 60), a variable was generated with four levels: (a) never case-level (*n* = 764); (b) No case-level incidence aged 60+ but previously case-level (*n* = 401); (c) case-level incidence aged 60+ and previously case-level (*n* = 220); (d) first incidence of case-level aged 60+ (*n* = 156) (Fig. 1).

### 2.4 Covariates

Consistent with previous analyses (Richards et al., 2014) the following variables were treated as potential confounders in additional models: sex, childhood occupational position, childhood cognitive ability (Hatch et al., 2007, Richards et al., 2001), adult occupational position and educational attainment (Opdebeeck et al., 2016, Richards et al., 2014); to investigate whether associations were those with fluid cognitive functions, and to further reduce the possibility of reverse causality, a measure of general cognitive ability, the National Adult Reading Test, was additionally adjusted (Nelson, 1991, Richards, 2003) (Fig. 1).

Childhood cognitive function at age 8 was represented as the sum of four tests of verbal and non-verbal ability devised by the National Foundation for Educational Research (Pigeon, 1964)**.** Childhood occupational position was derived from paternal occupation; adult occupational position was derived from participants’ own occupation at 53 years, given that this is when most people are expected to be in work, or earlier than this if information was missing. Occupational position was coded according to the UK Registrar General into six categories: professional, managerial, intermediate, skilled manual, semi-skilled manual and unskilled. The highest educational or training qualification achieved by 26 years was classified according to the Burnham scale and grouped into five categories: no qualification, below ordinary secondary qualifications (e.g., vocational qualifications), ordinary level qualifications (‘O’ levels or their training equivalents), advanced level qualifications (‘A’ levels or their equivalents) or higher education (degree or equivalent) (Hatch et al., 2009). Preliminary analysis revealed that sex x lifetime affect interactions were non-significant at the 5% level for all fully adjusted models; sex was therefore used as a covariate rather than stratifying variable. The National Adult Reading Test was administered at age 53, according to standard procedure (Nelson, 1991) but for scaling consistency and direction with other cognitive test outcomes, the conventional error-based score was reversed (max = 50) (Richards and Sackers, 2003).

To assess possible attenuating associations of anxiolytic and antidepressant medication, any (yes/no) use of anxiolytic (British National Formulary section 4.1.2) or antidepressant (British National Formulary section 4.3) medication at ages 36, 43, 53, 60–64, 69 was used as an additional adjustment (British National Formulary (March Issue), 2011). Information of medication use in adolescence was not available.

### 2.5 Statistical analyses

Only those with complete exposure and cognitive outcome data at age 69 were included in these analyses and therefore must have survived to age 69; covariates did not need to be complete. For each of the 3 cognitive tests separately, maximum likelihood estimated multivariable linear regression analyses were conducted to investigate whether case-level affective categories, and incidence affective categories, were associated with lower cognitive function at age 69. The predictor variable was entered as categorical, with never case-level as the reference group (Richards et al., 2014). For these analyses, Model 1 represents unadjusted associations; Model 2 adjusts for childhood cognition, childhood occupational position, educational attainment, adult occupational position and sex; Model 3 further adjusts for the National Adult Reading Test as a measure of general cognitive ability.

In addition, we ran a complementary analysis using a structured modelling approach to examine the best fit of different hypothesised life course models by which affective symptoms could be related to later life cognitive function (Table 3). To reduce multi-collinearity of repeated measures and ensure adequate statistical power, categories at three age spans were chosen to represent case-level affective symptoms over the life course: adolescence (ages 13–15), adulthood (ages 36–53) and later-life (ages 60–69). This was especially important to take into account the adolescent period in the current study. The approach is outlined in greater detail elsewhere (39). Briefly, it compares the model fit of a set of nested life course models (i.e., accumulation or risk of affective symptoms across the life course, or a sensitive period of exposure to affective symptoms in either adolescence, midlife or later adulthood) with a saturated model containing all main associations and all interactions. A *p*-value that is not statistically significant (*p* > 0.05) indicates no evidence that the more complex model explained the data better than the simpler life course model (more information is given in Supplementary IV). Results are shown as mean difference in cognitive scores with 95% confidence intervals; adjustments were not made for multiple comparisons in line with previous studies (Rothman, 1990).

### 2.6 Sensitivity analyses

First, as our previous analysis found no clear associations between longitudinal affective trajectories between ages 13 and 53, estimated by latent class analysis, and cognitive function at age 60–64, we re-ran the analysis using the case-level method up to age 53, instead of the latent class profiles, to check whether these null findings were explained by this different method of symptom capture (Richards et al., 2014). Second, the main cumulative analyses were repeated additionally adjusting for anxiolytic and antidepressant medication use. Third, the main cumulative analyses were re-run excluding participants with potentially clinically significant cognitive impairment (using the clinically validated ACE-III 82 threshold (Hsieh et al., 2013)); 81 study members (6.3%) fell below this threshold. Fourth, the main cumulative analyses were re-run adjusting for disease burden at age 69 (heart failure, angina, myocardial infarction, hyper/hypotension, stroke, diabetes, transient ischemic attacks, cancer, chronic lung disease, asthma, osteoarthritis, rheumatoid arthritis, osteoporosis, serious eye trouble, epilepsy, Parkinson's disease, memory problems and kidney disease at age 69). Fifth, another level was added to our grouping of affective episodes into: (a) never case-level; (b) once only; (c) twice only; (d) three times or more (Supplementary Table IX). Sixth, the main cumulative analyses were re-run using case-level symptoms up to age 60–64 with cognition at age 60–64 (Supplementary X).”

## 3 Results

In total, 1269 participants had complete data for all waves of affective symptoms and all cognitive outcomes, and 1541 participants had complete data for affective symptoms, verbal memory and search processing tests (but not ACE-III scores). Those without cognitive scores at age 69 were more likely to be male, have less than advanced educational attainment, and to have lower cognitive test scores at age 8 (all *p* < 0.01). For participants included in this analysis, compared with those with no evidence of affective problems across the lifetime, those with at least one case-level problem were more likely to be female and have less than advanced educational attainment (*p* < 0.01). Supplementary Table III shows mean cognitive test scores at age 69 by frequency of case-level symptoms.

### 3.1 Associations between lifetime case-level affective symptoms and cognitive function

Before covariate adjustment, those with case-level symptoms at only one assessment did not differ in cognitive function at age 69, compared with those with no case-level symptoms. Those with case-level symptoms at two or more assessments had lower ACE-III scores (unadjusted *β* = −1.08, 95% confidence interval (CI) = −1.90, −0.25), lower verbal memory (*β* = −0.63, 95% CI = −1.43, −0.17), letter search speed (*β* = −11.18, 95% CI = −20.65, −1.70) and accuracy (*β* = −0.92, 95% CI = −1.60, −0.25) at age 69 years (Table 1). With adjustments for sex, childhood cognition, childhood and midlife occupational position, educational attainment and measures of general cognitive ability, those with symptoms reaching case-level at only one assessment had lower letter search accuracy (*β* = −0.75, 95% CI = −1.38, −0.12), and those with two or more assessments had lower scores for all cognitive variables (Table 1). Most estimates were strengthened from unadjusted to adjusted models, implying negative confounding associations within unadjusted models.

**Table 1:** Regression coefficients representing associations between life course case-level affective categories and cognitive function at 69 years.

	Model 1		Model 2		Model 3	
	Cognitive scores, mean difference (95% CI)	*p*	Cognitive scores, mean difference (95% CI)	*p*	Cognitive scores, mean difference (95% CI)	*p*
**ACE-III scores**						
Never case-level (reference)						
Once case-level	0.04 (−0.68, 0.77)	0.91	−0.47 (−1.14, 0.20)	0.17	−0.58 (−1.23, 0.08)	0.08
≥2 times case-level	**−1.08 (−1.90, −0.25**)	**0.01**	**−0.91 (−1.68, −0.13**)	**0.02**	**−1.00** (**−1.75, −0.24**)	**0.01**
**Verbal memory**						
Never case-level (reference)						
Once case-level	0.23 (−0.47, 0.93)	0.52	−0.43 (−1.08, 0.23)	0.20	−0.45 (−1.10, 0.21)	0.18
≥2 times case-level	**−0.63 (−1.43, −0.17)**	**0.05**	**−1.22** (**−1.98, −0.45**)	**<0.01**	**−1.19** (**−1.94, −0.43**)	**<0.01**
**Letter search speed**						
Never case-level (reference)						
Once case-level	−7.03 (−15.36, 1.30)	0.10	−8.72 (−17.73, 0.29)	0.06	−8.16 (−17.24, −0.92)	0.08
≥2 times case-level	**−11.18 (−20.65, −1.70**)	**0.02**	**−14.07** (**−24.50, −3.65**)	**0.01**	**−14.42** (**−24.95, −3.89**)	**0.01**
**Letter search accuracy**						
Never case-level (reference)						
Once case-level	−0.53 (−1.12, 0.06)	0.08	**−0.75** (**−1.38, −0.13**)	**0.02**	**−0.75** (**−1.38, −0.12**)	**0.02**
≥2 times case-level	**−0.92 (−1.60, −0.25**)	**0.01**	**−1.08** (**−1.81, −0.36**)	**<0.01**	**−1.15** (**−1.88, −0.42**)	**<0.01**

Model 1 shows unadjusted coefficients; Model 2 shows coefficients adjusted for sex, childhood cognition, childhood occupational position, educational attainment, midlife.

With regard to symptom timing, the strongest effect sizes were found for those with case-level symptoms across the life course, followed by case-level incidence after age 53 for the ACE-III and memory. Case-level symptoms up to age 53 were negatively associated with letter search speed and accuracy with adjustments (Table 2).

**Table 2:** Regression coefficients representing associations between temporal incidence of case-level affective symptoms and cognitive function scores at 69 years.

	Model 1		Model 2		Model 3	
	Cognitive scores, mean difference (95% CI)	*p*	Cognitive scores, mean difference (95% CI)	*p*	Cognitive scores, mean difference (95% CI)	*p*
**ACE-III scores**						
Never case-level (reference)						
Case-level in earlier-life only	−0.04 (−0.73, 0.80)	0.93	−0.33 (−1.06, 0.40)	0.37	−0.27 (−0.98, 0.43)	0.45
Case-level in late-life and earlier-life	**−1.36** (**−2.31, −0.42**)	**0.01**	**−1.11** (**−2.00, −0.23**)	**0.01**	**−1.30** (**−2.16, −0.44**)	**0.01**
Case-level in late-life only	−0.21 (−1.26, 0.85)	0.70	−0.71 (−1.65, 0.22)	0.14	**−1.02** (**−1.93, −0.11**)	**0.03**
**Verbal memory**						
Never case-level (reference)						
Case-level in earlier-life only	0.38 (−0.36, 1.11)	0.31	−0.35 (−1.05, 0.35)	0.33	−0.27 (−0.97, 0.43)	0.45
Case-level in late-life and earlier-life	**−0.74 (−1.65, −0.17)**	**0.05**	**−1.23** (**−2.10, −0.37**)	**0.01**	**−1.23** (**−2.09, −0.37**)	**0.01**
Case-level in late-life only	−0.50 (−1.55, 0.55)	0.35	**−0.95** (**−1.89, −0.02**)	**0.05**	**−1.14** (**−2.07, −0.21**)	**0.02**
**Letter search speed**						
Never case-level (reference)						
Case-level in earlier-life only	−7.27 (−15.99, 1.44)	0.10	**−10.44 (−20.10, −0.78)**	**0.03**	**−9.98 (−19.71, −0.24)**	**0.05**
Case-level in late-life and earlier-life	**−14.04** (**−24.85, −3.23**)	**0.01**	**−17.37** (**−29.28, −5.46**)	**<0.01**	**−17.00** (**−29.00, −4.99**)	**0.01**
Case-level in late-life only	−4.85 (−17.30, 7.60)	0.45	−3.89 (−16.75, 8.96)	0.55	−4.40 (−17.36, 8.56)	0.51
**Letter search accuracy**						
Never case-level (reference)						
Case-level in earlier-life only	−0.64 **(−1.26, −0.02)**	0.08	**−0.88 (−1.56, −0.21)**	**0.01**	**−0.86 (−1.53, −0.18)**	**0.01**
Case-level in late-life and earlier-life	**−0.93** (**−1.70, −0.16**)	**0.01**	**−1.12** (**−1.95, −0.29**)	**0.01**	**−1.20** (**−2.03, −0.36**)	**0.01**
Case-level in late-life only	−0.47 (−1.36, 0.41)		−0.61 (−1.50, 0.28)	0.18	−0.68 (−1.57, 0.22)	0.14

Model 1 shows unadjusted coefficients; Model 2 shows coefficients adjusted for sex, childhood cognition, childhood occupational position, educational attainment, midlife occupational position; Model 3 shows coefficients further adjusted for a measure of general cognition, NART. NB: CI = confidence intervals. *p* < 0.05 notated in bold.

Late-life (60+) case-level incidence affect categories were defined as: late life incidence = case-level symptoms at assessments when participants were aged 60–64 and 69; earlier-life incidence = case-level symptoms at assessments when participants were aged < 60 (13, 15, 26, 36, 43, 53). The variable was recoded with four levels: (a) never case-level (reference); (b) no case-level incidence at age 60+ but previously case-level; (c) case-level incidence at age 60+ and previously case-level; (d) first incidence of case-level at 60+).

The results of the structured approach revealed that accumulation models and sensitive period models fitted the data as well as the saturated model with accumulation models being the best fit for 3 outcomes as they had the highest *p*-value (Table 3).

**Table 3:** Results of *p*-values for partial *f*-tests, comparing each represented life course models to a saturated model.

	**ACE-III**		**Verbal memory**		**Letter search speed**		**Letter search accuracy**	
Hypothesis	*f*	*p*	*f*	*p*	*f*	*p*	*f*	*p*
Saturated model	Reference		Reference		Reference		Reference	
No effect	2.42	0.02	1.86	0.04	2.11	0.03	1.86	0.04
Accumulation	**0.91**	**0.49**	**1.63**	**0.13**	**0.82**	**0.55**	**0.91**	**0.49**
Time period								
*t*1 (ages 13–15)	2.81	<0.01	**1.78**	**0.11**	**1.19**	**0.31**	**1.51**	**0.17**
*t*2 (ages 36–53)	**1.96**	**0.07**	**1.45**	**0.19**	**1.12**	**0.36**	**1.72**	**0.07**
*t*3 (ages 60–69)	**1.46**	**0.19**	**1.37**	**0.22**	**0.98**	**0.44**	**1.27**	**0.27**
Best model*	Accumulation		Sensitive period t3 (ages 60–69)		Accumulation		Accumulation	

To reduce multi-collinearity of repeated measures, categories at three age spans were chosen to represent case-level affective symptoms in the life course; adolescence (ages 13–15), adulthood (ages 36–53) and later-life (ages 60–69). A higher P-value (or lower *f* statistic) for the life course model equals a better model fit. The ‘saturated model’ is the most complicated model that contains affective case-level symptoms at all three ages, all two-way interactions and the three-way interaction. The ‘accumulation’ model proposes that the impact of exposure is cumulative over the life course and that the longer an individual is exposed to case-level symptoms, the greater the adverse impact on cognitive function at age 69. A ‘time period’ model proposes that exposure to case-level symptoms during a particular stage in life (e.g., *t*1, *t*2 or *t*3) has an adverse effect on cognitive function at age 69 with little or no influence of exposure to symptoms outside this specified time period. Bold values indicate *p*-value > 0.05.

*The best model in this case was determined by the lowest *f* value.

### 3.2 Sensitivity analyses

When we re-ran analyses for case-level cumulative symptoms and cognition up to ages 60–64, we replicated our previous null findings using longitudinal latent class profiles of symptoms up to age 53 (Supplementary Table V).

Findings were essentially unchanged when models were estimated with additional adjustments for life course history of anxiolytics or antidepressant medication use (Supplementary Table VI), and excluding those with ACE-III scores < 82 (Supplementary Table VII). After adjustment for disease burden (Supplementary Table VIII), and when another level was added to the grouping of affective episodes, changing groupings from three to four levels, the relationship between those with case-level problems and cognitive measures were slightly attenuated, but the pattern remained the same. Models using case-level symptoms up to age 60–64 provided evidence that frequency of symptoms was associated with lower verbal memory at age 60–64, but none of the other cognitive tests (Supplementary X).

## 4 Discussion

### 4.1 Main findings

In this population-based birth cohort study we investigated the association between prospectively reported affective problems across 50 years and cognitive function at age 69. Those with case-level affective problems at only one time point did not differ in cognitive function, compared with those without any case-level affective problems. However, those with case-level affective problems at two or more assessments across the life course had lower cognitive state (ACE-III), and lower verbal memory and letter search speed and accuracy at age 69, after adjusting for childhood cognition, childhood and adult occupational position, educational attainment, and general cognitive ability. Overall, our results suggest a cumulative negative effect of case-level affective problems on these outcomes. Life course persistence of symptoms was most strongly associated with the outcomes, with some additional evidence of symptom proximity for verbal memory.

### 4.2 Strengths and limitations

Major strengths of this study are: (1) use of a large population-representative birth cohort with over 50 years of follow-up since affective symptoms were first assessed in adolescence; (2) further measures of symptoms across this interval; (3) a range of cognitive outcomes including the most comprehensive measure of global cognitive state available; and (4) a range of potential confounders including cognitive function tested in childhood. Limitations to be considered are, first, like most longitudinal health surveys, there was a disproportionate loss to follow-up of those who were socioeconomically disadvantaged and had lower childhood cognitive scores, and we only included survivors. However, there is no reason to anticipate that this would have changed the pattern of associations observed. Second, although to some extent necessarily so, the measures of affective symptoms differed over time in NSHD. However, the thresholds for case-level symptoms were either clinically validated, or, in the case of the adolescent assessments, consistent with a previous percentile-based cut for the most severe symptoms (Richards and Abbott, 2009). However, the lack of clinical validation for the adolescent measures should be taken into consideration. Third, we did not take account of sub-threshold depressive symptoms in these analyses, which may have contributed to the associations observed. Fourth, we cannot rule out reverse causality since depressive symptoms were not captured between study assessments; thus cognitive decline may have triggered any intervening symptoms. Fifth, while we adjusted for anxiolytic and antidepressant medication, other medication could have confounded later-life cognition.

### 4.3 Interpretation

In a nationally representative birth cohort we found an association between cumulative case-level affective symptoms and diminished cognitive function in early old age. This is in line with our initial hypothesis: more lifetime affective problems – across over 50 years – would be associated with lower cognitive function in older age; and is consistent with shorter follow up studies suggesting a dose-response with increased frequency and severity of symptoms in relation to cognitive impairment (Köhler et al., 2010, Panza et al., 2009, 2010), and dementia (Chen et al., 2008, Dotson et al., 2010, Kaup et al., 2016, Saczynski et al., 2010). Our study highlights that this association is cumulative across the life course, with some evidence for the importance of proximal symptoms. Continuing follow-up of this cohort will determine whether recurrent symptoms ultimately raise the risk of clinical dementia. By controlling for childhood cognition and general cognitive ability in midlife our study goes some way towards reducing bias from reverse causation (Hatch et al., 2007, Scult et al., 2017). More detailed studies are required to distinguish whether affective symptoms are part of a causal chain leading to outcomes; are a prodromal aspect of dementia; or are marking longstanding physiological common cause.

Our results are in line with studies which find that slowing processing speed is particularly vulnerable in those with late-life depression (Brailean et al., 2008, Bunce et al., 2014, Butters et al., 2004, Sheline et al., 2006, van den Kommer et al., 2013). Our findings are also consistent with a meta-analysis (Diniz et al., 2013) and other studies (Butters et al., 2004, Singh-Manoux et al., 2017) demonstrating that those with late-life depression are at greater risk of cognitive impairment and dementia.

Notably, the present associations at age 69 were not observed in NSHD at ages 60–64 (Richards et al., 2014). This cannot be the result of changing the exposure definition from longitudinal latent classes to the frequency of case-level symptoms, since associations up to age 60–64 were not evident when reanalysed by the latter method. For at least two reasons this is more likely due to the association emerging by the subsequent wave of data at age 69. First, although a slight degree of cognitive decline was observed in NSHD as early as midlife (Richards et al., 2004), this decline has accelerated by age 69 (Davis et al., 2017). Second, the introduction of a comprehensive test of cognitive state at this age may have additionally captured cognition impairment of potential clinical significance. We also found some evidence that case-level symptoms incident after age 53 were associated with the outcomes, although, as noted, this may partially reflect reverse-causality.

While many potential behavioural and biological mechanisms linking depression to subsequent cognitive impairment and dementia have been proposed (Byers and Yaffe, 2011, Diniz et al., 2013), the pathways are not yet clearly understood and it is likely that multiple pathophysiological processes interact (Butters et al., 2008, Byers and Yaffe, 2011, Sweet et al., 2004). First, there is mounting evidence that vascular disease is associated with both depression and dementia, and ischemic damages to frontostriatal brain regions could be a common etiological factor (Butters et al., 2008, Sheline et al., 2008). A second proposed link is altered glucocorticoid production; this often occurs in affective disorders (Wolkowitz et al., 2010) and has been associated with atrophy of the hippocampus (Rothman and Mattson, 2010), a pathological feature associated with severe depression (McKinnon et al., 2009) and dementia (Barnes et al., 2009). A third proposed mechanism is a key feature observed in Alzheimer's disease, of increased accumulation of amyloid-β (Aβ) plaques (Rowe et al., 2010); initial work has indicated that Aβ is also increased in individuals with a lifetime history of depression (Wu et al., 2014), although one study did not observe this in individuals with late-life depression (De Winter et al., 2017). Other proposed pathophysiological processes include inflammatory pathways and impairments of nerve growth factors (Byers and Yaffe, 2011). It is plausible that pathological processes accumulate earlier, or to a greater extent, in those with lifetime affective problems, than in those without lifetime affective problems. Future work in NSHD using neuroimaging outcomes to identify functional and structural indicators associated with lifetime affective symptoms and cognitive aging may further elucidate processes underlying this relationship (Byers and Yaffe, 2011, Lane et al., 2017, Opdebeeck et al., 2016, Yasuno et al., 2016). Finally, we should note the attenuating effect of adjusting analyses for an index of disease burden; however, given the comprehensive nature of this index it is difficult to isolate any particular mechanism responsible. Further work to explore the possible role of physical disorders as part of a causal chain or marking common causes linking symptoms and cognition would be valuable.

Our findings are consistent with evidence that affective problems are a risk factor for lower cognitive function in later life (Gallagher et al., 2016, Pietrzak et al., 2015). They indicate a window in early old age when this risk becomes manifest. Continued follow-up will reveal the extent to which this risk translates into clinically significant outcomes. Meanwhile, our findings raise the possibility that effective management of affective problems across the life course may reduce this risk.

## Declarations of interest

None.

## Conflict of interest

All authors report no conflict of interest.

## Appendix Supplementary materials

Image, application 1
