# Supplementary material for: Lifetime affective problems and later-life cognitive state: Over 50 years of follow-up in a British birth cohort study
Source: J Affect Disord. 2018 Dec 1;241:348–55. doi: 10.1016/j.jad.2018.07.078 (PMC6137547; doi:10.1016/j.jad.2018.07.078)
Supplement: Supplementary file 1 [file mmc1.docx]

# Supplementary Online Content

1. **Supplementary Figure I.** Flow diagram of target samples for the postal questionnaire at age 68 and home visit at age 69 (amended from Kuh et al. 2016).
2. **Supplementary Table I.** Affective measures across the life course.
3. **Supplementary Table II.** Frequency of case-level affective problems*: A) cross sectional frequency of case-level symptoms and B) cumulative frequency of case-level symptoms at every wave across the life course.
4. **Supplementary Table III:** Means and standard deviations of the cognitive test scores at 69 years by case-level affective categories 13-69 years.
5. **Supplementary Table IV.** A structured approach to modelling the effects of binary exposure variables across the life course.
6. **Supplementary Table V.** Regression coefficients representing associations between life course case-level affective categories (between age 13-53) and cognitive function at 60 years.
7. **Supplementary Table VI.** Regression coefficients representing associations between life course case-level affective categories and cognitive function scores at 69 years, with model 3 plus adjustments for any anxiolytic or antidepressant medication.
8. **Supplementary Table VII.** Regression coefficients representing associations between life course case-level affective categories and cognitive function scores at 69 years, with model 3 but excluding those with ACE<82.
9. **Supplementary Table VIII.** Regression coefficients representing associations between life course case-level affective categories and cognitive function scores at 69 years, with model 3 plus adjustments for disease burden.
10. **Supplementary Table IX.** Regression coefficients representing associations between 4-level life course case-level affective categories and cognitive function scores at 69 years.
11. **Supplementary Table X.** Regression coefficients representing associations between 4 case-level affective categories up to age 60-64 and cognitive function scores at 60-64 years.

#
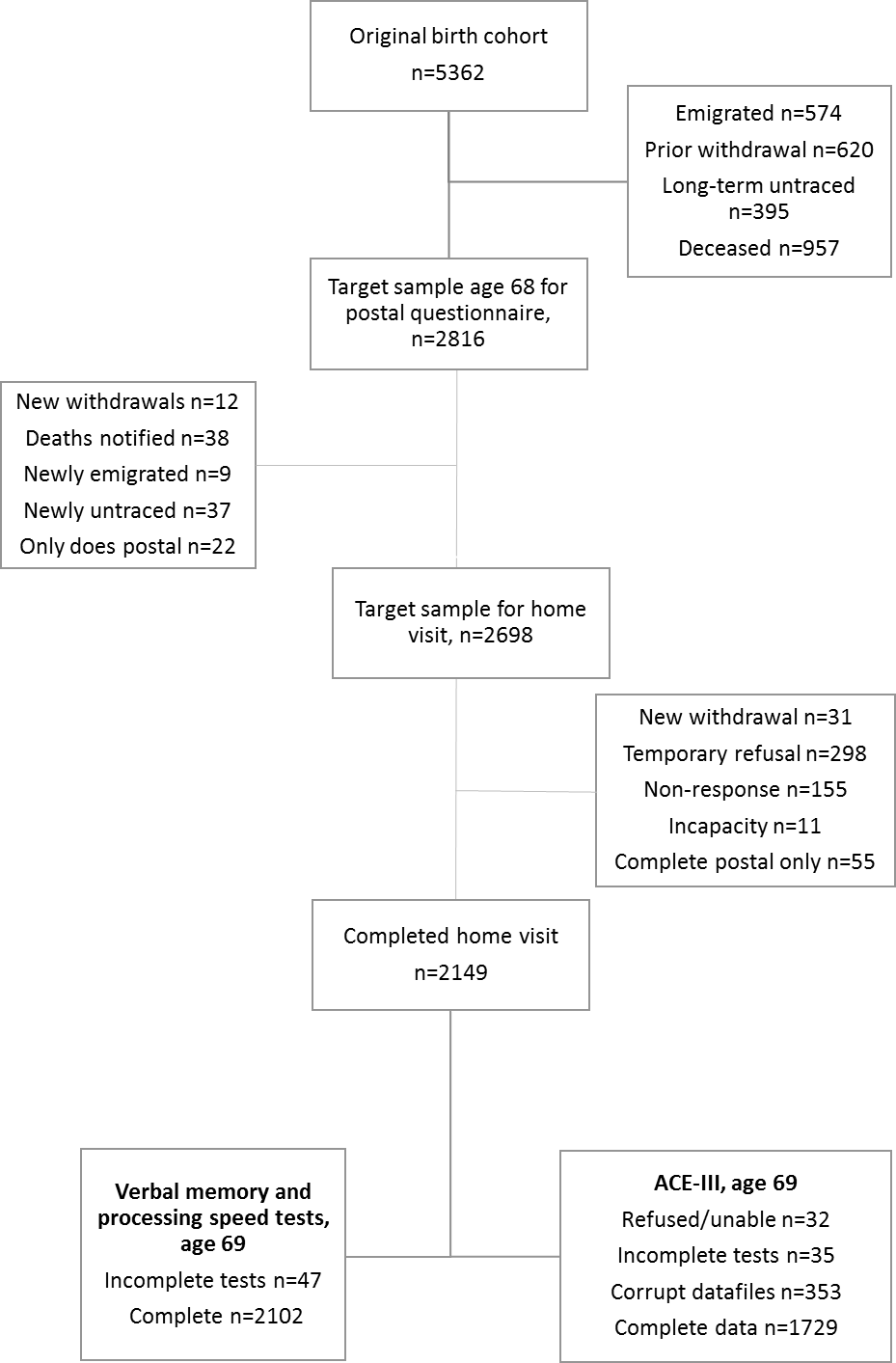
Supplementary Figure I: Flow diagram of target samples for the postal questionnaire at age 68 and home visit at age 69 (amended from Kuh et al. 2017).^1^

**Supplementary Table I:** **Affective measures across the life course.**

| **Year** | **Age** | **Measure** | **Threshold for case-level symptoms** | **Citations for generating case-level thresholds** | **Anti-depressant**  **/Anxiolytic Medication use in sample used in the analysis*** |
| --- | --- | --- | --- | --- | --- |
| 1959-1961 | 13-15 | Adolescent Rutter scale factor scores | 91st-100th percentile | Rutter et al. 1967 | - |
| 1982 | 36 | Present State Examination (PSE) short version | Index of definition≥5 | Wing et al. 1974  Rodgers and Mann 1986 | 47 (4%) |
| 1989 | 43 | Psychiatric Symptom Frequency scale (PSF) | ≥23 | Lindelow et al. 1997 | 51 (4%) |
| 1999 | 53 | 28-item General Health Questionnaire (GHQ-28) | ≥5 score (top 16%) | Goldberg et al. 1979, 1998 | 59 (5%) |
| 2006-2010 | 60-64 | 28-item General Health Questionnaire (GHQ-28) | ≥5 score (top 16%) | Goldberg et al. 1979, 1998 | 109 (9%) |
| 2015 | 69 | 28-item General Health Questionnaire (GHQ-28) | ≥5 score (top 16%) | Goldberg et al. 1979, 1998 | 105 (8%) |

*Self-reported use of anxiolytic (British National Formulary section 4.1.2) or antidepressant (British National Formulary section 4.3) medication.

**Year 1959-1961; Age 13/15: Teacher-rated questionnaire**

Affective symptoms were assessed by teachers observations of students’ anxious and depressive symptoms at age 13 and 15, using a forerunner of the Rutter A questionnaire ^2^. Teachers described aspects of the children’s personality, behavior, and attitudes on a 3-point scale—more, same, or less than classmates. These questionnaires have previously been subjected to exploratory factor analysis ^3,4^.with one factor consisting of items identified as anxiety/depression or internalizing emotions and behaviours such as “very anxious”, “Always tired and washed-out”, “frightened of rough games”, “extremely fearful”, “avoids attention”, “usually gloomy and sad”, “timid child”, “unable to make friends”, “diffident about competing” and “unduly miserable or worried about criticism”. Factor scores as derived by Xu et al., (2013) ^5^, at age 13 and 15 were summed and standardised to create a single measure of adolescent affective symptoms. These factor scores have been previously used in multiple subsequent analyses^3,5–9^.

**Year 1982; Age 36: Present-State-Examination (PSE):**

At age 36 affective symptoms were assessed using the short-form of the Present-State-Examination (PSE)^10^, which was administered by trained nurses to study members in their home. The PSE is a semi-structured clinical interview designed to assess the frequency and severity of psychiatric symptoms in the preceding month, and has shown good reliability between psychiatrists and non-psychiatrists if given appropriate training^11^. Each item is scored on a 3-point scale; symptom not present (0); moderate severity or frequency of symptom (1); severe and frequent symptom (2). A computer-generated, previously validated categorical variable was created from this 48-item diagnostic assessment through an index of definition (ID) where 5 or higher was taken as evidence of common mental disorder ^10^(see ^11^Rodgers and Mann (1986) for further details on the criterion-related validity of the PSE in this sample).

**Year 1989; Age 43: The Psychiatric Symptom Frequency Scale (PSF)**

Trained research nurses captured mental health at age 43 years by administering the Psychiatric Symptom Frequency Scale (PSF) scale containing a 19-item scale measuring current and recent (past 12months) depression and anxiety symptoms, and is based largely on questions from the Present-State-Examination ^6^. The scale has previously been subjected to exploratory factor analysis which revealed a single predominant factor incorporating symptoms of depression and anxiety, with high internal consistency (^12^). Receiver operating curve (ROC) analyses demonstrated that high scores on the PSF were strongly associated with suicidal ideation, and reports of contact with a health professional and use of medication for “nervous or emotional trouble or depression”. The analysis suggested cut offs of 13/14 or 22/23, depending on whether the scale was to be used for screening or defining a population at high risk of mental health disorder, respectively ^12,13^. Therefore in this analysis, we used a cut-off score of 23 or greater as a conservative estimate of individuals with high risk of an affective disorder ^12^.

**Year 1999-2015; Age 53, 60 and 69: The 28-item General Health Questionnaire (GHQ-28)**

The General Health Questionnaire (GHQ) is a self-reported screening questionnaire which assesses anxiety and depressive symptoms in the preceding four weeks, and is commonly used as a screening tool for psychiatric disorder in both primary care and community settings ^14^. Affective symptoms at age 53, 60 and 69 were measured using the 28-item version of this (GHQ-28), which consists of four sub-scales: somatic symptoms, anxiety and insomnia, social dysfunction and severe depression. Each item is rated on a four-point scale with answers ranging from 'better/healthier than normal', 'same as usual', 'worse/more than usual' to 'much worse/more than usual'. The GHQ can be scored in different ways depending on the research question. The standard ‘GHQ-scoring’ method scores each item 0-0-1-1 and is recommended as the best method for identifying ‘caseness’ ^14^. In contrast the Likert method scores each item 0-1-2-3, and is recommend when assessing the severity of affective symptoms. The standard GHQ score is the sum of each item, which ranges from 0-28 for the GHQ-scoring method (0-0-1-1). The overall validity and reliability of the 28-item GHQ has shown to be very high for both the GHQ-scoring and Likert scoring methods ^14^. At age 53, 60 and 69, severe case-level affective symptoms were defined by using the established and validated GHQ cut-point threshold of 5 or more symptoms ^14^.

# Supplementary Table II. Frequency of case-level affective problems*: A) cross sectional frequency of case-level symptoms and B) cumulative frequency of case-level symptoms at every wave across the life course.

| 1. Cross sectional affective problems | N | % | New cases |
| --- | --- | --- | --- |
| Case-level problem at age 13-15 | 288 | 18 | 288 |
| Case-level problem at age 36 | 78 | 5 | 61 |
| Case-level problem at age 43 | 140 | 9 | 92 |
| Case-level problem at age 53 | 289 | 19 | 180 |
| Case-level problem at age 60-64 | 265 | 17 | 103 |
| Case-level problem at age 69 | 206 | 13 | 53 |
|  |  |  |  |
| 1. Cumulative affective problems | N | % | Affective categories |
| 0 case-level problem across life course | 764 | 50 | 764 (50%) |
| 1 case-level problem across life course | 464 | 30 | 464 (30%) |
| 2 case-level problem across life course | 200 | 13 | 313 (20%) |
| 3 case-level problem across life course | 63 | 4 |  |
| 4 case-level problem across life course | 37 | 2 |  |
| 5 case-level problem across life course | 13 | 1 |  |
| 6 case-level problem across life course | 0 | 0 |  |

*****Data shown for participants with non-missing cognitive data at age 69.

# Supplementary Table III: Means and standard deviations of the cognitive test scores at 69 years by case-level affective categories 13-69 years.

|  | *N (%) for other cognitive scores* | *N (%) for ACE-III* | *ACE-III total*  *Mean (SD)* | *Verbal memory*  *Mean (SD)* | *Letter search speed*  *Mean (SD)* | *Letter search accuracy*  *Mean (SD)* |
| --- | --- | --- | --- | --- | --- | --- |
| Never case-level | 764 (50%) | 631 (50%) | 94.04 (5.59) | 22.47 (5.88) | 266.37 (72.60) | 24.05 (5.15) |
| Once case-level | 464 (30%) | 379 (30%) | 92.08 (5.66) | 22.70 (6.17) | 259.34 (74.35) | 23.52 (5.28) |
| >2 times case-level | 313(20%) | 259 (20%) | 90.96 (6.06) | 21.84 (6.10) | 255.19 (5.83) | 23.13 (4.76) |

NB: ACE-III=Third edition of the Addenbrooke’s Cognitive Examination. SD=standard deviation.

# Supplementary IV: A structured approach to modelling the effects of binary exposure variables across the life course.

Analyses were undertaken using STATA version 14. This analysis compromises of a structured modelling approach to examine different hypothesised life course affective models in relation to later-life cognitive function. The approach is outlined in greater detail in Mishra et al. 2009.

Briefly, the approach compares the model fit of a set of nested life course models with a saturated model containing all possible main effects and interaction. The original paper (Mishra et al. 2009), and subsequent papers (Murray et al. 2015, Jones et al. 2016), use the F-test to compare the fit of each reduced model compared to the saturated model. A p value that is not statistically significant (p>0.05), indicates that there is no evidence that the more complex model explained the data better than the simpler life course model. To reduce multi-collinearity of repeated measures, categories at three age spans were chosen to represent case-level affective symptoms in the life course; adolescence (ages 13-15), adulthood (ages 36-53) and later-life (ages 60-69), resulting in 8 possible trajectories. In the main analysis, the life course models analysed were as follows: 1) accumulation of risk models of affective case-level symptoms across all three time points. 2) sensitive period models for adolescence, adulthood or later-life.

**Table IV: All possible binary outcomes across three time points** (n=2093)**.**

|  |  |  |  |  |
| --- | --- | --- | --- | --- |
| Adolescence, age 13,15 | Adulthood, age 36-53 | Later-life, age 60-69 | n | n % |
| 0 | 0 | 0 | 1045 | 50.0 |
| 1 | 0 | 0 | 246 | 12.0 |
| 0 | 1 | 0 | 237 | 11.0 |
| 0 | 0 | 1 | 199 | 10.0 |
| 1 | 1 | 0 | 76 | 4.0 |
| 1 | 0 | 1 | 63 | 3.0 |
| 0 | 1 | 1 | 170 | 8.0 |
| 1 | 1 | 1 | 57 | 3.0 |

# Supplementary Table V. Regression coefficients representing associations between life course case-level affective categories (between age 13-53) and cognitive function at 60 years (n=1541).

As our previous analysis found no clear associations between longitudinal affective trajectories between ages 13 to 53, estimated by latent class analysis, and cognitive function at age 60-64, we re-ran this analysis using the case-level method up to age 53, instead of the latent class profiles, to explore whether these null findings were explained by this different method of symptom capture (12).

|  | Model 1 |  |
| --- | --- | --- |
|  | Cognitive scores,  Mean Difference (95% CI) | *p* |
| ***Verbal memory at age 60-64*** |  |  |
| Never case-level (reference) |  |  |
| Once case-level | -0.04 (-0.71,0.64) | 0.92 |
| ≥2 times case-level | 0.38 (-0.68,1.43) | 0.48 |
| ***Letter search speed at age 60-64*** |  |  |
| Never case-level (reference) |  |  |
| Once case-level | -9.94 (-17.94,1.93) | 0.09 |
| ≥2 times case-level | -5.43 (-17.91,7.06) | 0.39 |
| ***Letter search accuracy at age 60-64*** |  |  |
| Never case-level (reference) |  |  |
| Once case-level | -0.66 (-1.23,0.09) | 0.08 |
| ≥2 times case-level | -0.68 (-1.57,0.21) | 0.13 |

Model 1 shows unadjusted coefficients. NB: CI=confidence intervals. p<0.05 notated in bold.

# Supplementary Table VI. Regression coefficients representing associations between life course case-level affective categories and cognitive function scores at 69 years, with adjustments for any anxiolytic or antidepressant medication use, in addition to factors controlled for in model 3.

|  | Model 3 and medication | |
| --- | --- | --- |
|  | Cognitive scores,  Mean Difference (95% CI) | p |
| **ACE-III scores** |  |  |
| Never case-level (Reference) |  |  |
| Once case-level | -0.57 (-1.22,0.09) | 0.09 |
| ≥2 times case-level | **-0.97** (**-1.75,-0.19**) | **0.02** |
|  |  |  |
| **Verbal memory** |  |  |
| Never case-level (Reference) |  |  |
| Once case-level | -0.33 (-0.99,0.32) | 0.32 |
| ≥2 times case-level | **-0.84 (-1.62,-0.05)** | **0.04** |
|  |  |  |
| **Letter search speed** |  |  |
| Never case-level (Reference) |  |  |
| Once case-level | -7.89 (-17.02,1.25) | 0.09 |
| ≥2 times case-level | **-13.58 (-24.55,-2.61)** | **0.02** |
|  |  |  |
| **Letter search accuracy** |  |  |
| Never case-level (Reference) |  |  |
| Once case-level | **-0.72 (-1.35,-0.08)** | **0.03** |
| ≥2 times case-level | **-1.04 (-1.80,-0.28)** | **0.01** |

To assess the attenuating effects of anxiolytic and antidepressant medication, any use of anxiolytic (British National Formulary section 4.1.2) or antidepressant (British National Formulary section 4.3) medication at ages 36, 43, 53, 60-64, 69 was indicated (yes/no) and used as an additional adjustment.

Model 3 shows coefficients adjusted for sex, childhood cognition, childhood occupational position, educational attainment, midlife occupational position, a measure of general cognition-NART. NB: CI=confidence intervals. p<0.05 notated in bold.

# Supplementary Table VII. Regression coefficients representing associations between life course case-level affective categories and cognitive function scores at 69 years, excluding those with ACE-III<82, in addition to factors controlled for in model 3.

|  | Model 3 (subsample n=1188) | |
| --- | --- | --- |
|  | Cognitive scores,  Mean Difference (95% CI) | p |
| **ACE-III scores** |  |  |
| Never case-level (Reference) |  |  |
| Once case-level | -0.56 (-1.07,0.06) | 0.06 |
| ≥2 times case-level | **-0.65** (**-1.25,-0.04**) | **0.04** |
|  |  |  |
| **Verbal memory** |  |  |
| Never case-level (Reference) |  |  |
| Once case-level | -0.50 (-1.15,0.15) | 0.13 |
| ≥2 times case-level | **-1.21 (-1.98,-0.44)** | **0.01** |
|  |  |  |
| **Letter search speed** |  |  |
| Never case-level (Reference) |  |  |
| Once case-level | -8.68 (-17.90,0.54) | 0.07 |
| ≥2 times case-level | **-12.70 (-23.66,-1.74)** | **0.02** |
|  |  |  |
| **Letter search accuracy** |  |  |
| Never case-level (Reference) |  |  |
| Once case-level | **-0.69 (-1.33,-0.05)** | **0.03** |
| ≥2 times case-level | **-1.07 (-1.83,-0.30)** | **0.01** |

The main analyses were re-run excluding participants with potentially clinically significant cognitive impairment (using the clinically validated ACE-III 82 threshold); 81 study members (6.3%) fell below this threshold. Model 3 shows coefficients adjusted for sex, childhood cognition, childhood occupational position, educational attainment, midlife occupational position, a measure of general cognition-NART. Model 3 with the subsample shows results for only those with ACE≥82. NB: CI=confidence intervals. p<0.05 notated in bold.

# Supplementary Table VIII. Regression coefficients representing associations between life course case-level affective categories and cognitive function scores at 69 years, controlling for disease burden in addition to factors controlled for in model 3.

|  | Model 3 and disease burden | |
| --- | --- | --- |
|  | Cognitive scores,  Mean Difference (95% CI) | p |
| **ACE-III scores** |  |  |
| Never case-level (Reference) |  |  |
| Once case-level | -0.48 (-1.13,0.17) | 0.14 |
| ≥2 times case-level | **-0.79 (-1.56,-0.02)** | **0.04** |
|  |  |  |
| **Verbal memory** |  |  |
| Never case-level (Reference) |  |  |
| Once case-level | -0.44 (-1.11,0.22) | 0.18 |
| ≥2 times case-level | **-1.14 (-1.93,-0.35)** | **0.01** |
|  |  |  |
| **Letter search speed** |  |  |
| Never case-level (Reference) |  |  |
| Once case-level | -7.28 (-16.56,2.01) | 0.12 |
| ≥2 times case-level | **-11.12 (-22.16,-0.07)** | **0.04** |
|  |  |  |
| **Letter search accuracy** |  |  |
| Never case-level (Reference) |  |  |
| Once case-level | **-0.66 (-1.31,-0.01)** | **0.04** |
| ≥2 times case-level | **-0.82 (-1.59,-0.05)** | **0.03** |

Disease burden is a count of the number of doctor diagnosed chronic diseases or disorders over the last ten years, reported by participants. The research nurse asked about disorders: heart failure, angina, myocardial infarction, hyper/hypotension, stroke, diabetes, transient ischaemic attacks, cancer, chronic lung disease, asthma, osteoarthritis, rheumatoid arthritis, osteoporosis, serious eye trouble, epilepsy, Parkinson’s disease, memory problems and kidney disease. On this scale, we distinguished individuals with 0, 1, 2 or 3 or more doctor diagnosed diseases.

Model 3 shows coefficients adjusted for sex, childhood cognition, childhood occupational position, educational attainment, midlife occupational position, a measure of general cognition-NART. NB: CI=confidence intervals. p<0.05 notated in bold.

# Supplementary Table IX: Regression coefficients representing associations between 4-level life course case-level affective categories and cognitive function scores at 69 years, in addition to factors controlled for in model 3.

|  | Model 3 | |
| --- | --- | --- |
|  | Cognitive scores,  Mean Difference (95% CI) | p |
| **ACE-III scores** |  |  |
| Never case-level (reference) |  |  |
| Once case-level | 0.04 (-0.68,0.77) | 0.91 |
| 2 times case-level | **-1.62 (-2.59,-0.64)** | **0.01** |
| ≥3 times case-level | -0.11 (-1.35,1.13) | 0.86 |
|  |  |  |
| ***Verbal memory*** |  |  |
| Never case-level (reference) |  |  |
| Once case-level | 0.23 (-0.47,0.93) | 0.52 |
| 2 times case-level | **-1.02 (-1.96,-0.07)** | **0.04** |
| ≥3 times case-level | -0.05 (-1.14,1.25) | 0.93 |
|  |  |  |
| ***Letter search speed*** |  |  |
| Never case-level (reference) |  |  |
| Once case-level | -7.03 (-15.36,1.30) | 0.10 |
| 2 times case-level | *-9.67 (-20.88,1.54)* | *0.09* |
| ≥3 times case-level | **-13.86 (-28.11,-0.39)** | **0.05** |
|  |  |  |
| ***Letter search accuracy*** |  |  |
| Never case-level (reference) |  |  |
| Once case-level | -0.53 (-1.12,0.06) | 0.08 |
| 2 times case-level | **-0.80 (-1.60,-0.01)** | **0.05** |
| ≥3 times case-level | **-1.14 (-2.15,-0.13)** | **0.03** |

Model 3 shows coefficients adjusted for sex, childhood cognition, childhood occupational position, educational attainment, midlife occupational position, a measure of general cognition-NART. NB: CI=confidence intervals. p<0.05 notated in bold.

**Supplementary Table X.** **Regression coefficients representing associations between 4 case-level affective categories up to age 60-64 and cognitive function scores at 60-64 years.**

|  | Model 3 | |
| --- | --- | --- |
|  | Cognitive scores,  Mean Difference (95% CI) | p |
| **Verbal memory at age 60-64** |  |  |
| Never case-level (Reference) |  |  |
| Once case-level | -0.46 (-1.03,1.07) | 0.12 |
| ≥2 times case-level | **-1.19** (**-1.93,-0.45**) | **0.03** |
|  |  |  |
| **Letter search speed at age 60-64** |  |  |
| Never case-level (Reference) |  |  |
| Once case-level | -4.51 (-12.64,3.61) | 0.27 |
| ≥2 times case-level | 3.05 (-7.49,13.60) | 0.57 |
|  |  |  |
| **Letter search accuracy at age 60-64** |  |  |
| Never case-level (Reference) |  |  |
| Once case-level | -0.48 (-1.02,0.06) | 0.10 |
| ≥2 times case-level | -0.16 (-0.85,0.54) | 0.66 |

Model 3 shows coefficients adjusted for sex, childhood cognition, childhood occupational position, educational attainment, midlife occupational position, a measure of general cognition-NART. NB: CI=confidence intervals. p<0.05 notated in bold.

**References**

1. Kuh D, Wong A, Shah I, et al. The MRC National Survey of Health and Development reaches age 70: maintaining participation at older ages in a birth cohort study. *Eur J Epidemiol*. 2016;31(11):1135-1147. doi:10.1007/s10654-016-0217-8.

2. Rutter M, Tizard J, Whitmore K. *Education, Health and Behaviour.* Longman Publishing Group.; 1970.

3. Colman I, Murray J, Abbott RA, et al. Outcomes of conduct problems in adolescence: 40 year follow-up of national cohort. *BMJ*. 2009;338.

4. Rodgers B. Behaviour and Personality in Childhood as Predictors of Adult Psychiatric Disorder. *J Child Psychol Psychiatry*. 1990;31(3):393-414. doi:10.1111/j.1469-7610.1990.tb01577.x.

5. Xu MK, Jones PB, Barnett JH, et al. Adolescent self-organization predicts midlife memory in a prospective birth cohort study. *Psychol Aging*. 2013. doi:10.1037/a0033787.

6. Hatch SL, Mishra G, Hotopf M, Jones PB, Kuh D. Appraisals of stressors and common mental disorder from early to mid-adulthood in the 1946 British birth cohort. *J Affect Disord*. 2009;119(1):66-75. doi:10.1016/j.jad.2009.03.021.

7. Colman I, Ploubidis GB, Wadsworth MEJ, Jones PB, Croudace TJ. A longitudinal typology of symptoms of depression and anxiety over the life course. *Biol Psychiatry*. 2007;62(11):1265-1271. doi:10.1016/j.biopsych.2007.05.012.

8. Paykel ES, Hayhurst H, Abbott R, Wadsworth  M. Stability and change in milder psychiatric disorder over 7 years in a birth cohort. *Psychol Med*. 2017;31:1373-1384. doi:10.1017\S0033291701004652.

9. Van Os J, Jones PB. Early risk factors and adult person--environment relationships in affective disorder. *Psychol Med*. 1999;29(5):1055-1067.

10. Wing J, Cooper J, Sartorius N. Present State Examination. *Inst Psychiatry*. 1974.

11. Rodgers B, Mann S A. The Reliability and Validity of PSE Assessments by Lay Interviewers: A National Population Survey - Journals - NCBI. *Psychol Med*. 1986;16(3):689-700.

12. Lindelow M, Hardy R, Rodgers B. Development of a scale to measure symptoms of anxiety and depression in the general UK population: the psychiatric symptom frequency scale. *J Epidemiol Community Health*. 1997;51(5):549-557.

13. Archer G. Lifetime affective symptoms and mortality in the MRC National Survery of Health and Development. 2017.

14. Goldberg DP, Hillier VF. A scaled version of the General Health Questionnaire. *Psychol Med*. 1979;9(1):139-145.
